# Supplementary material for: Autologous iPSC-Derived Human Neuromuscular Junction to Model the Pathophysiology of Hereditary Spastic Paraplegia
Source: Cells. 2022 Oct 24;11(21):3351. doi: 10.3390/cells11213351 (PMC9655384; doi:10.3390/cells11213351)
Supplement: Supplementary file 1 [file cells-11-03351-s001.zip › cells-1960366-supplementary.pdf]

cells

Supplementary Materials

# Autologous iPSC-Derived Human Neuromuscular Junction to Model the Pathophysiology of Hereditary Spastic Paraplegia

## SUPPLEMENTARY FIGURE TITLES AND LEGENDS

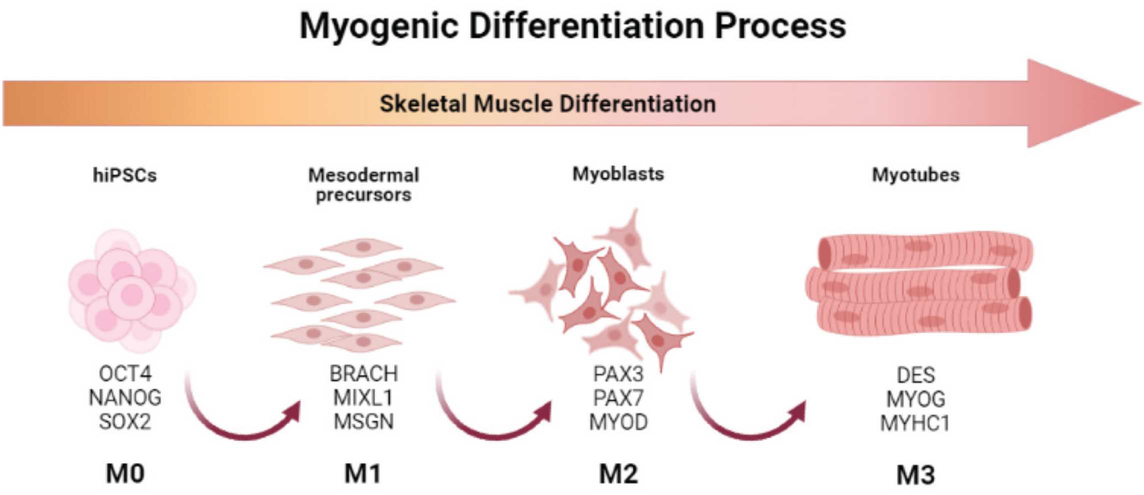


**Figure S1.** Myogenic differentiation process, related to figure 3. Schematic overview of the myogenic differentiation protocol for HSP and CTRL hiPSC lines towards myotubes. hiPSCs were kept pluripotent in M0 (E8 medium). Three different media were used to induce myogenic differentiation at three different steps: M1 to differentiate hiPSCs to the stage of mesodermal precursors, M2 to induce the myoblast stage, M3 to promote fusion of the myoblasts into myotubes. The qRT-PCR genes for checking the progression of the differentiation have been indicated. The protocol was adapted from Caron and colleagues (Caron et al., 2016).

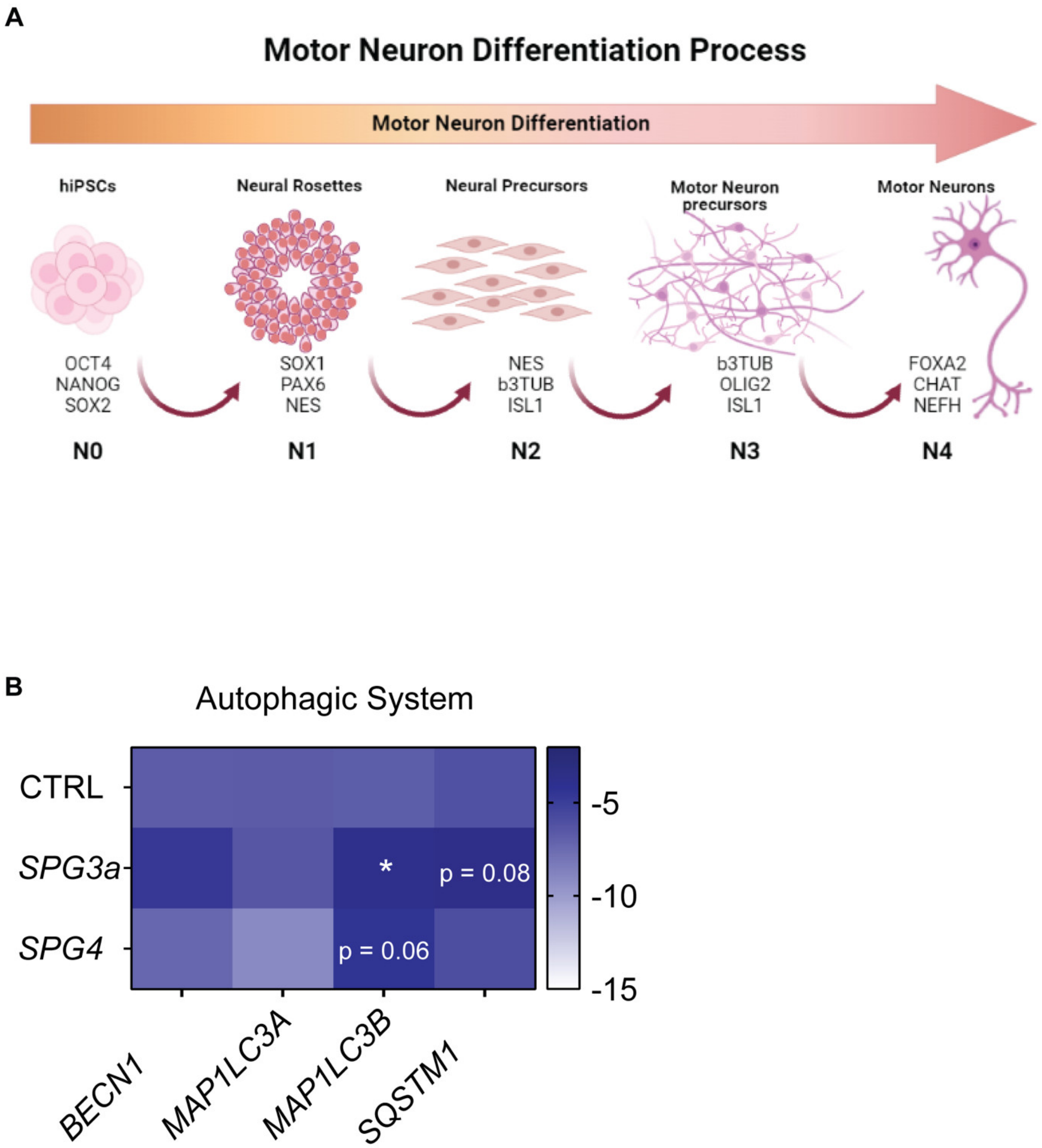


**Figure S2.** MN differentiation process, related to figure 4. (**A**) Schematic overview of the MN differentiation protocol for HSP and CTRL hiPSCs towards MNs. hiPSCs were kept pluripotent in N0 (E8 medium). Four distinct media were used to induce MN differentiation at four different steps: N1 to differentiate hiPSCs to the stage of neural rosettes, N2 to propagate the cells obtained, N3 to induce the MN precursors, N4 to obtain mature MNs. The qRT-PCR genes for checking the progression of the differentiation have been indicated. The protocol was adapted from Bianchi and colleagues (Bianchi et al., 2018). (**B**) Heat map for the genes regulating the autophagic proteolytic system (*BECN1, MAP1LC3A, MAP1LC3B* and *SQSTM1*) in CTRL and HSP-derived MNs. Each data point was represented as ⊚Ct, normalized for the housekeeping genes *(GAPDH* and *RPL13a)*. Data were representative of independent experiments and values were expressed as mean ± SEM. Significance of the differences for *SPG*-mutated vs. CTRL lines: *p < 0.05. For p < 0.1, the actual value was reported.

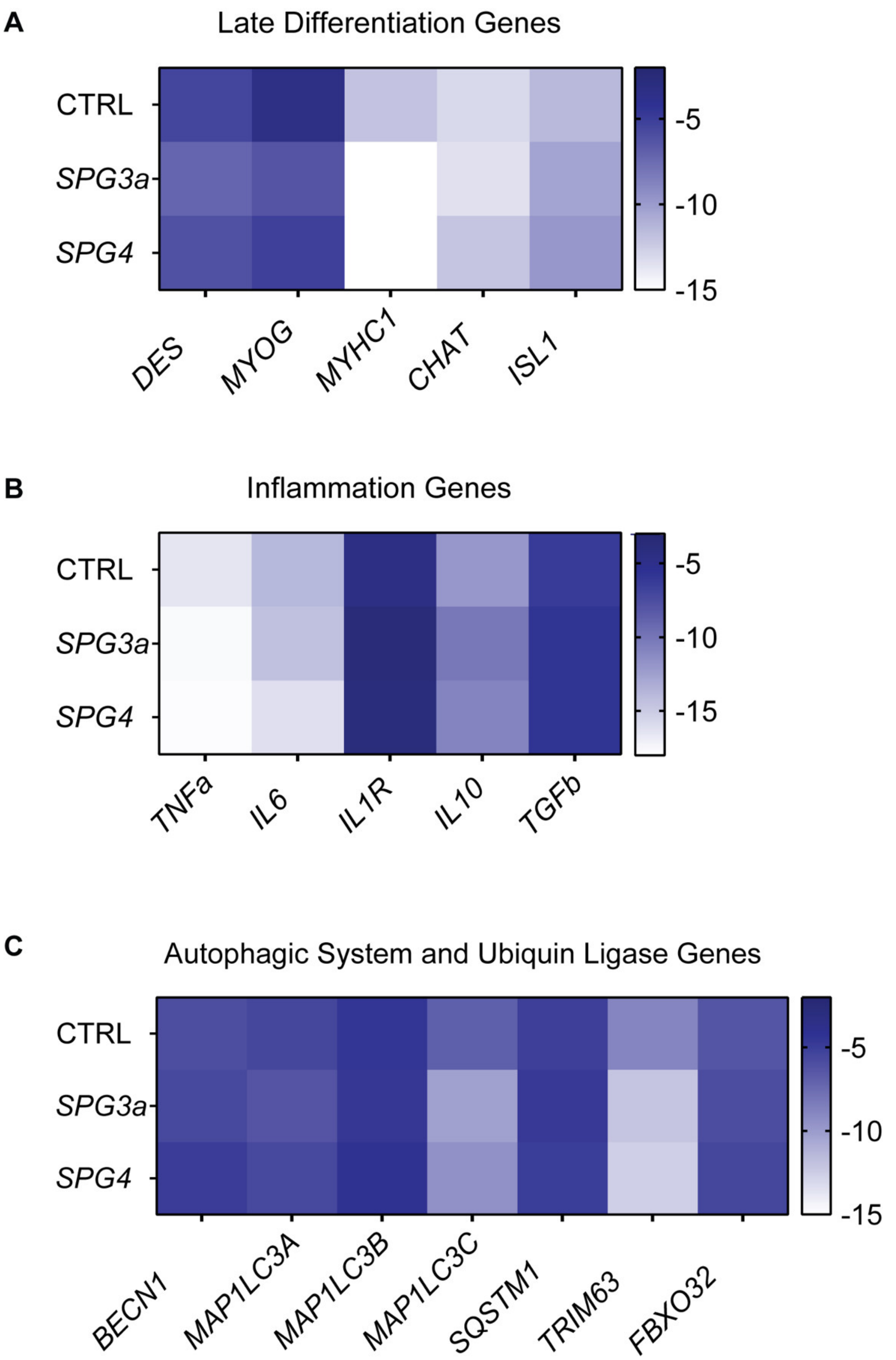


**Figure S3.** Gene expression profiles for NMJs, related to figure 6. Heat maps reporting genes regulating (**A**) the late-stage myogenic (*DES*, *MYOG* and *MYHC1*) and MN (*CHAT* and *ISL1*) differentiations, (**B**) inflammation (*TNFα*, *IL6*, *IL1R*, *IL10* and *TGFβ*) and (**C**) autophagic system (*BECN1*, *MAP1LC3A*, *MAP1LC3B*, *MAP1LC3C* and *SQSTM1*) or muscle specific E3 ubiquitin ligases (*TRIM63* and *FBXO32*). Each data point was represented as ΔCt, normalized for the housekeeping genes *(GAPDH* and *RPL13a)*. Data were representative of independent experiments and values were expressed as mean ± SEM.

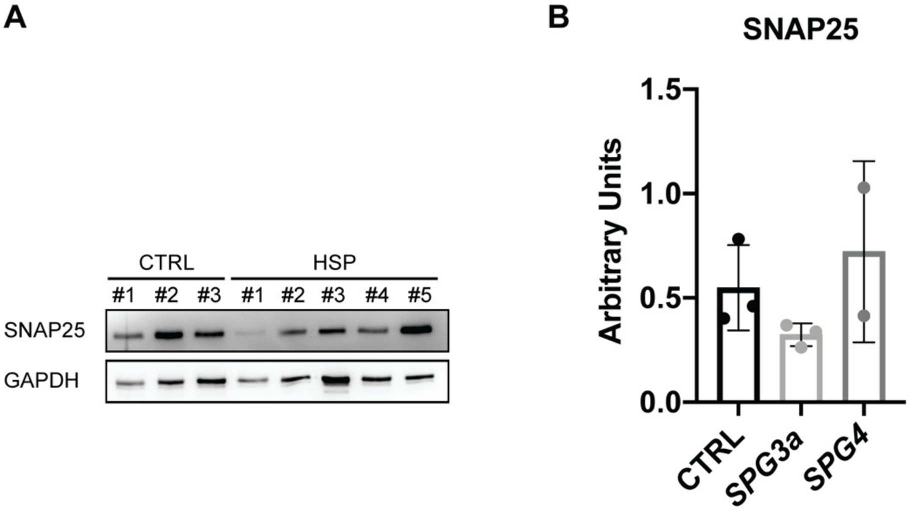


**Figure S4.** SNAP25 protein expression in NMJs, related to figure 6. WB analysis (**A**) and quantification (**B**) for the protein levels of SNAP25 in CTRL (three different hiPSC lines) and HSP-derived (#1, #2, #3 for *SPG3a*- and #4, #5 for *SPG4*-mutated genotypes) lines. Data were analyzed by one-way ANOVA followed by a Dunnett post hoc test. Data were representative of independent experiments and values were expressed as mean ± SD.

## SUPPLEMENTARY TABLES

**Table S1.** Genetic features of HSP patients, related to Experimental procedures (Study design and ethics statement).

| Patient | Gender | Inheritance | Gene | Variant DNA | Protein | Variant Protein | Zygosity | Disorder |
|---|---|---|---|---|---|---|---|---|
| **HSP #1** | F | AD | *SPG3a* | c.1483C > T (exon 12) | ATLASTIN1 | p.Arg495Trp | Heterozygous | *SPG3a*-HSP |
| **HSP #2** | M | AD | *SPG3a* | c.757G > A (exon 8) | ATLASTIN1 | p.Val253Ile | Heterozygous | *SPG3a*-HSP |
| **HSP #3** | M | AD | *SPG3a* | c.757G > A (exon 8) | ATLASTIN1 | p.Val253Ile | Heterozygous | *SPG3a*-HSP |
| **HSP #4** | M | AD | *SPG4* | c.1066G > A (exon 7) | SPASTIN | p.Glu356Lys | Heterozygous | *SPG4*-HSP |
| **HSP #5** | M | AD | *SPG4* | c.1496G > A (exon 13) | SPASTIN | p.Arg499His | Heterozygous | *SPG4*-HSP |

AD: autosomal dominant.

**Table S2.** Clinical parameters of HSP patients, related to Experimental procedures (Study design and ethics statement).

| | **Patient** | **HSP #1** | **HSP #2** | **HSP #3** | **HSP #4** | **HSP #5** |
|---|---|---|---|---|---|---|
| | **Age at onset (years)** | 2 | 1 | 3 | 1 | 1,5 |
| | **Age at diagnosis (years)** | 4,25 | 7 | 9 | 5 | 3 |
| **Lower limbs** | **Gait** | Pathologic | Pathologic | Pathologic | Pathologic | Pathologic |
| | **Muscle weakness** | yes | yes | yes | yes | yes |
| | **Spasticity** | yes | yes | yes | yes | yes |
| | **Hyperreflexia** | yes | yes | yes | yes | yes |

| | | | | | | |
|---|---|---|---|---|---|---|
| | **Muscle contractures** | yes | yes | yes | yes | yes |
| | **Ankle clonus** | yes | no | no | no | yes |
| | **Babiski sign** | yes | yes | yes | yes | yes |
| | **GMFCS** | II | I | I | I | III |
| **Treatment** | **Botulinum toxin** | 4x (last in 2018) | 3x (2003, 2005, 2012) | no | 1x (2014) | 1x (2012) |
| | **Surgery** | no | yes (2012) | no | no | yes (2012, 2014) |
| | **Others** | ADHD, dysarthria | no | no | Autism spectrum disorder | Intellectual disability |

ADHD: attention deficit hyperactivity disorder.

**Table S3.** List of primers for qRT-PCR, related to Experimental procedures (Quantitative Real-Time PCR (qRT-PCR) analysis).

| Gene | Primer Direction | Primer Sequence (5′ > 3′) |
|---|---|---|
| ***ACTA*** | forward | GAAGATTCGTCGTCCTGAGAA |
| | reverse | ACCACATGTGGATCACCAAG |
| ***ADAM22*** | forward | GAAGACGAAAGTCGGCACGA |
| | reverse | TGAATGACGTTCCAAAGGCAT |
| ***ANK2*** | forward | ACCTGCAATCAGAATGGACTCA |
| | reverse | TGCAATGTGAAGAGCGGTATT |
| ***b3TUB*** | forward | CCTCCGTGTAGTGACCCTT |
| | reverse | GGCCTTTGGACATCTCTTCAG |
| ***BRACH*** | forward | ACCCAGTTCATAGCGGTGAC |
| | reverse | AAGCTTTTGCAAATGGATTG |
| ***BECN1*** | forward | AACAGCGTTTGTAGTTCTGACA |
| | reverse | GACTACGACTTGTGTAGCGTC |
| ***CASK*** | forward | TTGAAATCGTAAAGCGAGCTGA |
| | reverse | CAGTAGCGTAGAGCTTCCAGTA |
| ***CDX2*** | forward | GACGTGAGCATGTACCCTAGC |
| | reverse | GCGTAGCCATTCCAGTCCT |
| ***CHAT*** | forward | CCCTGATGCCTTCATCCA |
| | reverse | GTAGGTGGGCACCAGTCTTC |
| ***CHRNA1*** | forward | GATGAAGTAAATCAGATCGTGACAACC |
| | reverse | TTCACACCGCCATAGTCATCTGGATTCCAT |
| ***CHRNB1*** | forward | AGAATGGCCAGTGGGAGAATAT |
| | reverse | CTATTGGGTTTGGGGAAGAGAA |
| ***CHRNG*** | forward | GTCCCAGACTTACAGCACCAATG |
| | reverse | GATGAGGATGGCGACAGAGGAG |
| ***CHRND*** | forward | AACGGGGAGTGGGAGATAGTCCA |
| | reverse | CTTCTTGACCCCCTCAGACAGCA |
| ***CHRNE*** | forward | CTCTCAGACGTACAATGCCGAA |
| | reverse | GTGGCGCAGCCGCGGGGACATGG |
| ***c-MYC*** | forward | TCCTCGGATTCTCTGCTCTCCT |
| | reverse | AGAAGGTGATCCAGACTCTGACCT |
| ***CLTA*** | forward | CGATTGCAGTCAGAGCCTGAAAG |

| | | |
|---|---|---|
| | reverse | TAGCTGCTCGTCCTGTCTTGCA |
| ***CLTB*** | forward | CAACGGTCCTGCTGATGGCTAC |
| | reverse | GCCATTCCTGTTCCGTGACCTT |
| ***DES*** | forward | TCGGCTCTAAGGGCTCCTC |
| | reverse | CGTGGTCAGAAACTCCTGGTT |
| ***DLG1*** | forward | TCGAACTAGCCAGAAGCGATCC |
| | reverse | TGCCTGGCTTGCCACCATTCAT |
| ***DLG2*** | forward | GCCGGTGATTATCCTGGGG |
| | reverse | CGCTTTGGCCTCGTAGTATGA |
| ***DLG3*** | forward | TGGAGGTCAACCTGCTCAAAGG |
| | reverse | TAGGCGTCCATCCTTCTGAGCA |
| ***DLG4*** | forward | ACCGAGGCGAATTGTGATCC |
| | reverse | CCGTCTGACCCGCATTCTT |
| ***DNM1*** | forward | ATCGCCACTTGGCTGACCGTAT |
| | reverse | CCTCCTTCTCAATGGACAGTAGC |
| ***DNM2*** | forward | CGAGTCACTGTCCTGGTACAAG |
| | reverse | TGCTCCGTGTTGAAGATGGCGA |
| ***DNM3*** | forward | GCTCACCATCAGCAACATTGGC |
| | reverse | CCGAACTTTCAGGTTGTCCAAGG |
| ***DOC2B*** | forward | CACCAAGACCTTCAACATCTGCC |
| | reverse | GCCTTGCTTCTGTGAGCTGTAC |
| ***EN1*** | forward | CGCAGCAGCCTCTCGTATG |
| | reverse | CCTGGAACTCCGCCTTGAG |
| ***EOMES*** | forward | CTGCCCACTACAATGTGTTCG |
| | reverse | GCGCCTTTGTTATTGGTGAGTTT |
| ***FOXA2*** | forward | GGAGCAGCTACTATGCAGAGC |
| | reverse | CGTGTTCATGCCGTTCATCC |
| ***FOXO32*** | forward | GCCTTTGTGCCTACAACTGAA |
| | reverse | CTGCCCTTTGTCTGACAGAAT |
| ***GAPDH*** | forward | TCAAGAAGGTGGTGAAGCAGG |
| | reverse | ACCAGGAAATGAGCTTGACAAA |
| ***GATA4*** | forward | CGACACCCCAATCTCGATATG |
| | reverse | GTTGCACAGATAGTGACCCGT |
| ***GDF-3*** | forward | ACACCTGTGCCAGACTAAGATGCT |
| | reverse | TGACGGTGGCAGAGGTTCTTACAA |
| ***HAND2*** | forward | ATGAGTCTGGTAGGTGGTTTTCC |
| | reverse | CATACTCGGGGCTGTAGGACA |
| ***HB9*** | forward | CTTCTGTTTCTCCGCTTCCT |
| | reverse | CACCTCGCTCATGCTCAG |
| ***HOXC4*** | forward | ACGAGAAAGAGAGTGGGAGAGA |

| | | |
|---|---|---|
| | reverse | GGAGGTCTGGGGTTGAG |
| ***HPRT*** | forward | TGACACTGGCAAAACAATGCA |
| | reverse | GGTCCTTTTCACCAGCAAGCT |
| ***hTERT*** | forward | AAATGCGGCCCCTGTTTCT |
| | reverse | CAGTGCGTCTTGAGGAGCA |
| ***IL10*** | forward | GACTTTAAGGGTTACCTGGGTTG |
| | reverse | TCACATGCGCCTTGATGTCTG |
| ***IL1R*** | forward | ATGAAATTGATGTTCGTCCCTGT |
| | reverse | ACCACGCAATAGTAATGTCCTG |
| ***IL6*** | forward | ACTCACCTCTTCAGAACGAATTG |
| | reverse | CCATCTTTGGAAGGTTCAGGTTG |
| ***ISL1*** | forward | GCGGAGTGTAATCAGTATTTGGA |
| | reverse | GCATTTGATCCCGTACAACCT |
| ***KLF-4*** | forward | CGGACATCAACGACGTGAG |
| | reverse | GACGCCTTCAGCACGAACT |
| ***MAP1LC3A*** | forward | AACATGAGCGAGTTGGTCAAG |
| | reverse | GCTCGTAGATGTCCGCGAT |
| ***MAP1LC3B*** | forward | AAGGCGCTTACAGCTCAATG |
| | reverse | CTGGGAGGCATAGACCATGT |
| ***MIXL1*** | forward | CTGAGGAGCCATGACTGACA |
| | reverse | TGGGAGTGTGGGCTTAAAAC |
| ***MSGN*** | forward | CTGCACACCCTCCGGAATTA |
| | reverse | AGGAGGTCTGTGAGTTCCCC |
| ***MYHC1*** | forward | GACATTGACCACACCCAGTATAA |
| | reverse | CAGCTTCTCATCTCGCATCTC |
| ***MYOD*** | forward | CCGCCTGAGCAAAGTAAATG |
| | reverse | CGATATAGCGGATGGCGTT |
| ***MYOG*** | forward | CAGGCTCAAGAAGGTGAATGA |
| | reverse | CGATGTACTGGATGGCACTG |
| ***NANOG*** | forward | TGGCCGAAGAATAGCAATGGTGTG |
| | reverse | TTCCAGGTCTGGTTGCTCCACATT |
| ***NEFH*** | forward | GTGAAGAGTGTCGGATTGGCT |
| | reverse | ACACAGAGGGAATTTTGGGGA |
| ***NEFM*** | forward | ACAACCACGACCTCAGCAGCTA |
| | reverse | GTTGAGGAGGTCCTGGTATTCG |
| ***NES*** | forward | TCAGCTTTCAGGACCCCAAG |
| | reverse | TGGGAGCAAAGATCCAAGACG |
| ***NEUROD1*** | forward | CTGCTCAGGACCTACTAACAACAA |
| | reverse | GTCCAGCTTGGAGGACCTT |
| ***NEUROG2*** | forward | TCCTCTTCCTCCTTCAACTCC |
| | reverse | GCCAAAGTCACAGCAACG |
| ***NFL*** | forward | CCAAGACCTCCTCAACGTGAAG |
| | reverse | ATGCTTCCCACGCTGGTGAAAC |
| ***NKX2.5*** | forward | ACCTCAACAGCTCCCTGACTCT |
| | reverse | ATAATCGCCGCCACAAACTCTCC |
| ***OCT4*** | forward | CGAGCAATTTGCCAAGCTCCTGAA |
| | reverse | GCCGCAGCTTACACATGTTCTTGA |

| | | |
|---|---|---|
| ***OLIG2*** | forward | CACAGAGCAGTGGGGAGTG |
| | reverse | GCACACAGCGGTACCTTTTC |
| ***PAX3*** | forward | ATTAAGCCACACATGCCGGT |
| | reverse | TACACAAGGAAGCCCCTGCT |
| ***PAX6*** | forward | AACGATAACATACCAAGCGTGT |
| | reverse | GGTCTGCCCGTTCAACATC |
| ***PAX7*** | forward | GGGCCTCCTGCTTGTTTAT |
| | reverse | CCATCTGGCTGGACTTCAAT |
| ***REX1*** | forward | TGGAGGAATACCTGGCATTGACCT |
| | reverse | AGCGATTGCGCTCAGACTGTCATA |
| ***RPL13a*** | forward | CCTGGAGGAGAAGAGGAAAGAGA |
| | reverse | TTGAGGACCTCTGTGTATTTGTCAA |
| ***SHANK2*** | forward | CGTGGCAAGCCGGACTAAG |
| | reverse | AAGGACCAGGTGATTCCCTCC |
| ***SLC17A6*** | forward | GGGTTTTCGGAGCTGCCATA |
| | reverse | CACACCCTCAACAAGTCCCTG |
| ***SLC17A7*** | forward | CAGAGTTTTCGGCTTTGCTATTG |
| | reverse | GCGACTCCGTTCTAAGGGTG |
| ***SNAP25*** | forward | ACCAGTTGGCTGATGAGTCG |
| | reverse | CAAAGTCCTGATACCAGCATCTT |
| ***SOX1*** | forward | AAAGTCAAAACGAGGCGAGA |
| | reverse | AAGTGCTTGGACCTGCCTTA |
| ***SOX2*** | forward | TGGCGAACCATCTCTGTGGT |
| | reverse | CCAACGGTGTCAACCTGCAT |
| ***SOX17*** | forward | GTGGACCGCACGGAATTTG |
| | reverse | GGAGATTCACACCGGAGTCA |
| ***SQSTM1*** | forward | GACTACGACTTGTGTAGCGTC |
| | reverse | AGTGTCCGTGTTTCACCTTCC |
| ***STON1*** | forward | TATGAGAGTGCCTACCAGGCAG |
| | reverse | ACTGAACAGTAGCAAATGGATACC |
| ***STON2*** | forward | GTGTGGAGGATAAACCGACTGC |
| | reverse | CGTGATTGGCAAATCTGGAAGGC |
| ***STX1A*** | forward | TAAAGAGCATCGAGCAGTCCA |
| | reverse | GACATGACCTCCACAAACTTTCT |
| ***SVOP*** | forward | TGGACTCTGTACTATGGCATCC |
| | reverse | TGGCCCAGAATACCTCAATCA |
| ***SYN1*** | forward | TGCTCAGCAGTACAACGTACC |
| | reverse | GACACTTGCGATGTCCTGGAA |
| ***SYNDIG1*** | forward | ACACCCTGTCCTACGATGTG |
| | reverse | TCTCTGTGTCGCTTGAGTAGTC |
| ***SYP*** | forward | CTCGGCTTTGTGAAGGTGCT |
| | reverse | CTGAGGTCACTCTCGGTCTTG |
| ***SYT2*** | forward | CGTGGACAACTCCACTGAGAGT |

| | | |
|---|---|---|
| | reverse | CAACCACAGCAATGGCGATCAG |
| ***SYT3*** | forward | AGCCCTCCTACTTGGACATGG |
| | reverse | GGGATGTTTGGCTCGGTTTGA |
| ***SYT6*** | forward | TACGCTACGATTACGAGACCG |
| | reverse | GCTTCCACAAAAGTCCTTGGC |
| ***SYT12*** | forward | GTTCCCCAATTACGACTACAGG |
| | reverse | TCAATGCTGAGACTGCCTTTG |
| ***TBR1*** | forward | TCACTGGAGGTTTCAAGGAGGC |
| | reverse | TTTCTTGGCGCATCCAGTGAGC |
| ***TGF*[illegible]** | forward | CAATTCCTGGCGATACCTCAG |
| | reverse | GCACAACTCCGGTGACATCAA |
| ***TNF*[illegible]** | forward | CCTCTCTCTAATCAGCCCTCTG |
| | reverse | GAGGACCTGGGAGTAGATGAG |
| ***TRIM64*** | forward | CTTCCAGGCTGCAAATCCCTA |
| | reverse | ACACTCCGTGACGATCCATGA |
| ***VAMP1*** | forward | ACATGACCAGTAACAGACGACT |
| | reverse | ACGTTCACACGTATGATGTCC |

**Table S4.** List of antibodies for IF and WB, related to Experimental procedures (IF, Western blot).

| Protein | Antibody Name (#Catalog Number) | Provider | IF | WB |
|---|---|---|---|---|
| **ATL1** | ATL1 Monoclonal Antibody (1F6B12) (Rabbit monoclonal) **(#MA5-31641)** | Thermo Fisher Scientific | | 1:1000 |
| **ACTN2** | Anti-SARCOMERIC ALPHA ACTININ (EA-53) (Mouse monoclonal) **(#ab9465)** | Abcam | 1:200 | 1:500 |
| **GAPDH** | Anti-GAPDH Antibody (Rabbit polyclonal) **(G9545)** | Merck | | 1:1000 |
| **ISL1** | Recombinant Anti-ISLET 1 antibody (EP4182) - Neural Stem Cell Marker (Rabbit monoclonal) **(#ab109517)** | Abcam | 1:500 | 1:60 |
| **LIN28** | LIN-28 (S-15) (Goat polyclonal) **(#sc-54032)** | Santa Cruz Biotechnology | 1:50 | |
| **MF20** | MYHC1 (MF20) (Mouse monoclonal) | Development Studies Hybridoma Bank (DSHB) | 1:300 | 1:3 |
| **MYOD** | MYOD1 (D8G3) XP Rabbit mAb (Rabbit monoclonal) **(#13812)** | Cell Signaling | 1:300 | 1:1000 |
| **NANOG** | NANOG (Rabbit polyclonal) **(#PA1-097)** | Thermo Fisher Scientific | 1:200 | |
| **NEFH** | Anti-NEUROFILAMENT HEAVY POLYPEPTIDE antibody (Rabbit polyclonal) **(#ab8135)** | Abcam | 1:1000 | 1:100 |
| **NEST** | Purified anti-NESTIN Antibody (10C2) (Mouse monoclonal) **(#656801)** | BioLegend | | 1:250 |
| **OCT4** | Anti-OCT4 - Embryonic Stem Cell Marker (Goat polyclonal) **(#ab27985)** | Abcam | 1:200 | |
| **OCT4** | Anti-OCT4 antibody (Rabbit polyclonal) **(#ab19857)** | Abcam | | 1:150 |
| **PAX6** | PAX6 (D3A9V) XP Rabbit mAb (Rabbit monoclonal) **(#60433)** | Cell Signaling | 1:100 | 1:150 |
| **SOX2** | SOX-2 (Y-17) | Santa Cruz Biotechnology | 1:50 | |

| | | | | |
|---|---|---|---|---|
| | (Goat polyclonal) **(#sc-17320)** | | | |
| **SPAST** | Anti-SPASTIN antibody (Sp3G11/1)<br>(Mouse monoclonal) **(#ab31850)** | Abcam | | 1:200 |
| **SSEA4** | SSEA-4 (MC813)<br>(Mouse monoclonal) **(#sc-59368)** | Santa Cruz Biotechnology | 1:50 | |
| **TBA1B** | Acetyl-$\alpha$-TUBULIN (Lys40) (D20G3)<br>(Rabbit monoclonal) **(#5335)** | Cell Signaling | 1:500 | |
| **TRA-1-60** | TRA-1-60 (TRA-1-60)<br>(Mouse monoclonal) **(#sc-21705)** | Santa Cruz Biotechnology | 1:50 | |
| **TUBA4A** | Anti-◎-TUBULIN antibody (B-5-1-2)<br>(Mouse monoclonal) **(#T6074)** | Merck | | 1:1000 |
| **TUBB3** | Anti-beta III TUBULIN antibody (2G10) - Neuronal Marker<br>(Mouse monoclonal) **(#ab78078)** | Abcam | 1:500 | 1:300 |

**Supplementary Video S1**. related to figure 6. Phase-contrast video-micrograph of spontaneously contracting hiPSC-derived CTRL myotubes co-cultured with hiPSC-derived CTRL MNs at day 9.

**Supplementary Video S2**. related to figure 6. Phase-contrast video-micrograph of spontaneously contracting hiPSC-derived *SPG3a*-mutated myotubes co-cultured with hiPSC-derived *SPG3a*-mutated MNs at day 9.

**Supplementary Video S3**. related to figure 6. Phase-contrast video-micrograph of spontaneously contracting hiPSC-derived *SPG4*-mutated myotubes co-cultured with hiPSC-derived *SPG4*-mutated MNs at day 9.
